# Addressing Bias in Responder Analysis of Patient-Reported Outcomes

**DOI:** 10.1007/s43441-021-00298-5

**Published:** 2021-05-27

**Authors:** Joseph C. Cappelleri, Richard Chambers

**Affiliations:** 1 Global Biometrics and Data Management, Pfizer Inc., Groton, 445 Eastern Point Road, MS 8260-2502, Groton, CT 06340 USA; 2 Global Biometrics and Data Management, Pfizer Inc., Collegeville, PA USA

**Keywords:** Information bias, Patient-reported outcomes, Responder analysis, Misclassification, Measurement error, Treatment effect

## Abstract

**Introduction:**

Quantitative patient-reported outcome (PRO) measures ideally are analyzed on their original scales and responder analyses are used to aid the interpretation of those primary analyses. As stated in the FDA PRO Guidance for Medical Product Development (2009), one way to lend meaning and interpretation to such a PRO measure is to dichotomize between values where within-patient changes are considered clinically important and those that are not. But even a PRO scale with a cutoff score that discriminates well between responder and non-responders is fraught with some misclassification.

**Methods:**

Using estimates of sensitivity and specificity on classification of responder status from a PRO instrument, formulas are provided to correct for such responder misclassification under the assumption of no treatment misclassification. Two case studies from sexual medicine illustrate the methodology.

**Results:**

Adjustment formulas on cell counts for responder misclassification are a direct extension of correction formulas for misclassification on disease from a two-way cross-classification table of disease (yes, no) and exposure (yes, no). Unadjusted and adjusted estimates of treatment effect are compared in terms of odds ratio, response ratio, and response difference. In the two case studies, there was considerable underestimation of treatment effect.

**Discussion and conclusions:**

The methodology can be applied to different therapeutic areas. Limitations of the methodology, such as when adjusted cell estimates become negative, are highlighted. The role of anchor-based methodology is discussed for obtaining estimates of sensitivity and specificity on responder classification. Correction for treatment effect bias from misclassification of responder status on PRO measures can lead to more trustworthy interpretation and effective decision-making.

**Clinicaltrials.gov:** NCT00343200

## Introduction

Ideally, a clinical trial should be able to demonstrate not only a statistically significant improvement in a clinical endpoint, but also that the magnitude of the effect is clinically relevant. A patient-reported outcome (PRO) measure, one type of clinical endpoint, is any report on the status of a patient’s health condition that comes directly from the patient, without interpretation of the patient’s response by a clinician or anyone else [1]. Unlike well-established clinical measurements such as survival and blood pressure, which are generally understood and can be measured directly, the scoring of latent (unobserved) concepts captured by a PRO measure (and health measurement scales in general) may be unfamiliar to many healthcare professionals and patients [2]. Researchers may lack the sufficient data, experience, or clinical understanding to interpret the relevance or meaningfulness of scores or change in scores on a self-reported rating scale.

Quantitative PRO measures ideally are analyzed on their original metric and responder analyses are used to augment the interpretation of those primary analyses. One way to lend meaning and interpretation to a quantitative PRO measure is to dichotomize between values where within-patient changes are considered clinically important (“responders”) and those that are not [3, 4]. This type of responder analysis is in common use in clinical trials and has been described in regulatory documents [1, 5], especially where “soft” clinical endpoints such as PRO measures are used. For instance, interpretation can be enriched by establishing meaningful change in PRO measures at the individual level (i.e., defining a responder) and calculating and comparing the proportion of response as defined by this predetermined clinically important change between the treatment groups [1]. The procedure is useful because between-group differences in responder proportions or percentages may be understood more intuitively than a difference in mean scores from rating scales.

Consider, for instance, a PRO measure like self-reported pain measured with a 11-point pain intensity numerical rating scale, where 0 = no pain and 10 = worst possible pain, over the past 24 h [6]. One proposed approach to enrich its interpretation, and that of other patient-reported measures, is to conduct a responder analysis where a quantitative measure is dichotomized into "responders" and "non-responders" [7, 8]. The outcome can be defined as a score on the pain scale at a postbaseline visit or as change from baseline to the postbaseline visit. In this type of responder analysis, the original metric of pain can be dichotomized at a cutoff or threshold value one side of which a subject is considered a “responder” (e.g., at least 30% reduction or two-point absolute reduction in pain from baseline to end of study) and the other side of which a subject is considered to be a “non-responder” [6].

Anchor-based methods, which examine the association between the targeted concept of the PRO instrument and the concept measured by the anchor (or anchors), can provide the primary empirical evidence to estimate a cutoff or threshold score for the responder definition of the targeted PRO; distribution-based approaches, which rely solely on the distribution of the data using descriptive measures (such as means, standard deviations, reliability of the PRO measure), can serve as an adjunct method to determine a responder cutoff of the PRO measure [1, 2, 4, 9]. But classification of responder status, be it based on anchor-based or distribution-based approaches, is not measured perfectly. Even a PRO scale with a cutoff score that discriminates well between responders and non-responders is fraught with some misclassifications: Some individuals classified as responders (based on the cutoff or threshold score on the PRO measure) may in fact be non-responders; some individuals classified as non-responder may, in fact, be responders. Yet there has been no attempt in research to adjust for responder misclassification on a PRO measure.

In this article, formulas are provided to correct for responder misclassification under the assumption of no treatment misclassification in a two-by-two contingency table. A general framework is provided to illustrate how responder misclassification affects measures of treatment effect (responder ratio, responder difference, odds ratio). Estimates of treatment effect are compared between unadjusted and adjusted estimates of treatment effect using two cases studies from sexual medicine to illustrate the methodology. Limitations of the methodology are discussed. The article concludes with points to consider and possible extensions on responder analysis and misclassification.

## Methods: Correction Formulas for Responder Misclassification

Formulas exist for correcting for misclassification on disease or exposure, or both, for a two-way cross-classification table of disease status (yes, no) and exposure status (yes, no) [10, 11]. But these formulas have not been applied in the context of responder analysis in general and for PRO measures in particular. In the current exposition, no misclassification of treatment is assumed, a reasonable assumption in experimental and quasi-experimental studies where the investigator directs treatment allocation (be it randomly or non-randomly). The formulas with misclassification on disease only (and no misclassification on treatment) can be applied directly and modified by replacing disease (yes, no) with responder status (yes, no).

Suppose that a validation study is undertaken on responder status of the targeted patient-reported outcome of interest. Assume that data consist of observed responder status (yes, no) by self-report, and there was a way to know with certainty each subject’s true responder status. In this case, the data could be laid out as a two-by-two contingency table shown in Table 1, called a validation table. Along the interior columns, subjects are classified according to their true responder status, while along the interior, row subjects are classified as responder or non-responder according to the numerical threshold or cutoff chosen on the PRO measure of interest.

**Table 1:** Validation table

	True responder	True non-responder
Classified as responder	E	F
Classified as non-responder	G	H
Total	E + G	F + H

*Note* Letters (*E*, *F*, *G*, *H*) represent counts of subjects, sensitivity = *E*/(*E* + *G*), and specificity = *H*/(*F* + *H*)

Table 1 contains elements of diagnostic statistics that are used to correct measures for misclassification. In particular, the sensitivity of response is the number that are true responders who are classified correctly as such, divided by the number of true responders; the specificity of response is the number that are true non-responders who are classified correctly as such, divided by the number of true non-responders.

Table 2 provides the equations for calculating expected observed data from the true data given sensitivity and specificity, adapted from other sources based on disease misclassification [10, 11]. The equations in Table 2 can then be algebraically rearranged to solve, in reverse, for the true or corrected cell counts (*A*, *B*, *C*, *D*) as a function of the observed cell counts (*a*, *b*, *c*, *d*) and sensitivity and specificity for responder classification under the assumption of differential or non-differential misclassification with respect to treatment (Table 3). In this case, non-differential misclassification exists when the pair of sensitivities for responder classification, one for experimental treatment and the other for control treatment, are equal and, separately, when the pair of specificities for responder classification also do not differ between the two treatments; otherwise, when either pair or both pairs are different between treatments, differential misclassification exists.

**Table 2:** Equations for calculating expected observed data (when there is responder misclassification only): based on true data

Outcome	Truth		Expected observed	
	Treatment		Treatment	
Response	T1	T0	T1	T0
R+	A	B	*a* = *A(*SE_T1_) + *C*(1−SP_T1_)	*b* = *B*(SE_T0_) + *D*(1−SP_T0_)
R−	C	D	*c* = *C(*SP_T1_) + *A*(1−SE_T1_)	*d* = *D(SP*_T0_) + *B*(1−SE_T0_)
Total	A + C	B + D	*a* + *c*	*b* + *d*

**Table 3:** Equations for correcting observed data given sensitivity and specificity for responder misclassification (no treatment misclassification)

	Observed		Expected truth	
Outcome	Treatment		Treatment	
Response	T1	T0	T1	T0
R+	a	b	*A* = [*a*(SP_T1_)−*c*(1−SP_T1_)] ** ÷ **(SE _T1_ + SP _T1_−1)	*B* = [*b*(SP _T0_)−*d*(1 −SP _T0_)] **÷ **(SE_T0_ + SP _T0_−1)
R−	c	d	*C* = [*c*(SE_T1_)−*a*(1−SE _T1_)] **÷ **(SE _T1_ + SP_T1_−1)	*D* = [*d*(SE_T0_)−*b*(1 −SE_T0_)] **÷ **(SE _T0_ + SP_T0_−1)
Total	*a* + *c*	*b* + *d*	A + C	B + D

*Note* As with Table 1, T1 and T0 denote experimental treatment and control treatments, respectively, and are measured perfectly (no misclassification of treatment); R+ denotes responder, R− denotes non-responder; SE_T1_ and SE_T0_ are sensitivity of responder status in T1 and T0, respectively (which permit for differential misclassification); SP_T1_ and SP_T0_ are specificity of responder status in T1 and T0, respectively (which permit for differential misclassification). Tables 2 and 3 also allow for non-differential misclassification where, by definition, the mechanism of misclassification assumes that sensitivities and, separately, the specificities for misclassifying responder status do not differ by treatment (i.e., SE_T1_ = SE_T0_ = SE and SP_T1_ = SP_T0_ = SP) and the denominator therefore reduces to SE + SP−1

Again, instead of the outcome being disease status, as is commonly considered, the outcome now becomes responder status; typically, the other variable is also called the exposure variable that is referred to as the treatment variable here. As noted, responder status and treatment type are each taken to have two levels or categories.

## Effect of Non-differential Responder Misclassification on Estimates of Treatment Effect

**Fig. 1:**
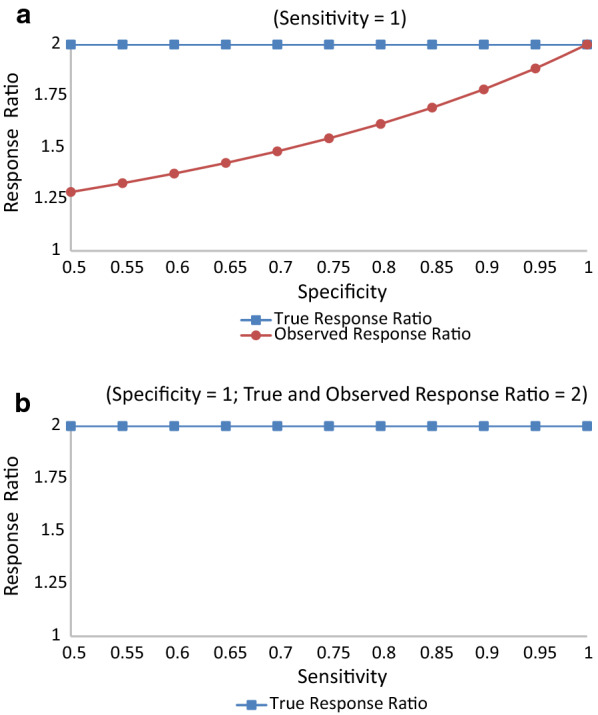
**a** Response Ratio vs. Specificity (Sensitivity = 1). **b**. Response Ratio vs. Sensitivity (Specificity = 1; True and Observed Response Ratio = 2)

**Fig. 2:**
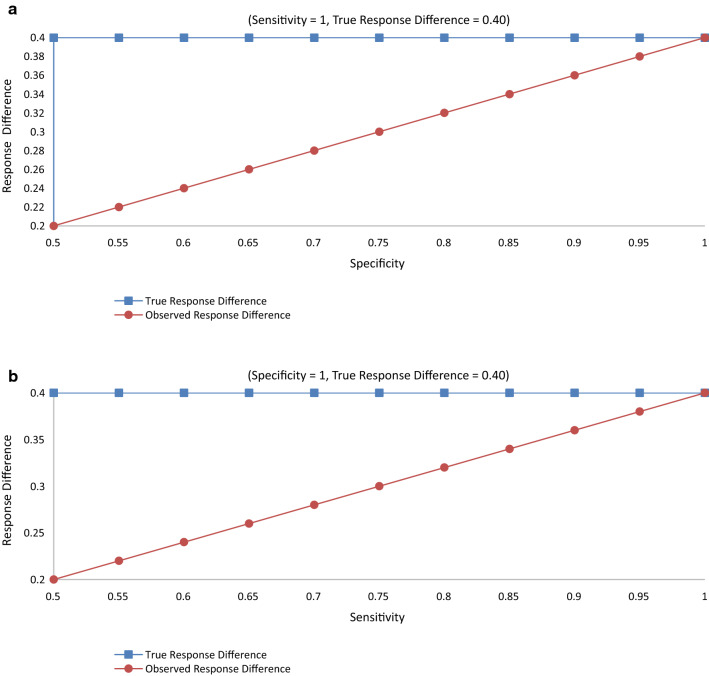
**a** Response Difference vs. Specificity (Sensitivity = 1, True Response Difference = 0.40). **b** Response Difference vs. Sensitivity (Specificity = 1, True Response Difference = 0.40)

**Fig. 3:**
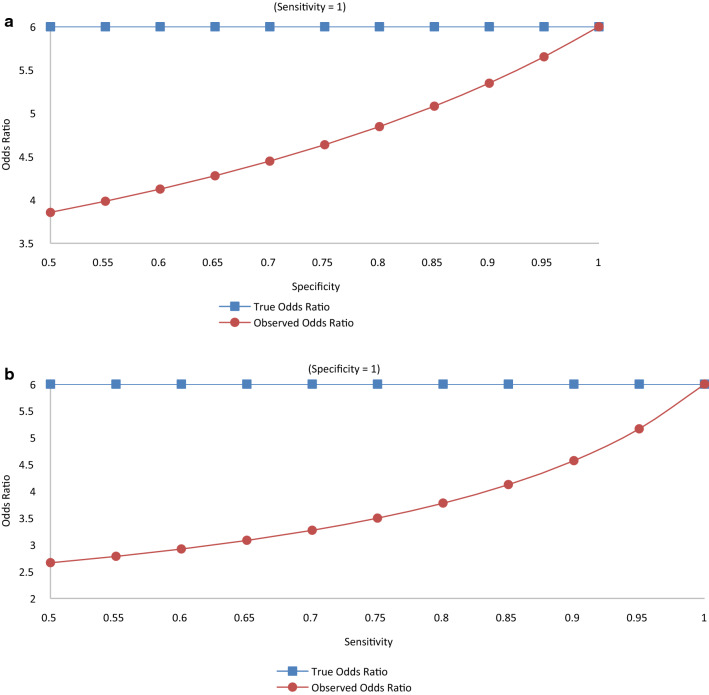
**a**. Odds Ratio vs. Specificity (Sensitivity = 1). **b**. Odds Ratio vs. Sensitivity (Specificity = 1)

In Table 4 data are presented for a hypothetical study with 2,000 subjects, half on the experimental treatment and the other half on the control treatment. The true association between treatment and response has a response ratio of 2, a response difference of 0.4, and an odds ratio of 6. With these data, Figs. 1, 2 and 3 show the relation of sensitivity and specificity with the expected observed response ratio (Fig. 1), response difference (Fig. 2), and odds ratio (Fig. 3) under the assumption of non-differential responder misclassification and no treatment misclassification. Alternative scenarios on treatment response rates and their consequences can be produced by directly applying the same formulas from Table 2 and, without loss of generality, would show the same general patterns as those depicted in Figs. 1, 2 and 3 (when the experimental treatment response exceeds the control treatment response).

**Table 4:** Hypothetical true association between treatment and response

Response status	Treatment status	
	Experimental	Control
Responder (R^+^)	*A* = 800	*B* = 400
Non-responder (R^−^)	*C* = 200	*D* = 600
Total	1000	1000
Response rate	0.8	0.4
Response difference	0.4	
Response ratio	2.0	
Odds ratio	6.0

When sensitivity is held at 100% and specificity ranges from 50 to 100%, there is an inverse relation between specificity and bias in the response ratio; as the specificity increases, the bias decreases (Fig. 1a). Even a specificity of 95% will yield a response ratio of 1.88, close to the truth (2.0) though not completely unbiased. Conversely, when specificity is held at 100% and sensitivity ranges from 50 to 100%, the analysis yields the unbiased response ratio of 2 regardless of the sensitivity (Fig. 1b).

When sensitivity is held at 100% and specificity ranges from 50 to 100%, the response difference approaches the truth linearly as the specificity approaches 100% (Fig. 2a). In this situation, the response difference can be corrected by dividing the observed response difference by the specificity, which is then expected to equal the true response difference [11]. In like fashion, when specificity is held at 100% and sensitivity ranges from 50 to 100%, the response difference approaches the truth linearly as the sensitivity approaches 100% (Fig. 2b). Here, the response difference can be corrected by dividing the observed response difference by the sensitivity to obtain the expected truth.

When sensitivity is held at 100% and specificity ranges from 50 to 100%, the odd ratio approaches the truth somewhat linearly as the specificity approaches 100% (Fig. 3a). In contrast, when specificity is held at 100% and sensitivity ranges from 50 to 100%, the odds ratio is further from the truth and exponentially approaches the truth as sensitivity approaches 100% (Fig. 3b).

## Two Case Studies from a Clinical Trial

As an illustration of the methodology, a post hoc analysis was undertaken on data from a randomized, double-blind, placebo-controlled, flexible-dose, sildenafil citrate (Viagra) trial in which men were randomized to receive sildenafil or placebo for 12 weeks [12]. This trial was conducted in accordance with Good Clinical Practice Guidelines and the Declaration of Helsinki, and was approved by local institutional review boards. All subjects provided written informed consent before enrollment.

From this clinical trial, two illustrative examples of post hoc responder analyses were performed on the six-item erectile function domain of the International Index of Erectile Function (IIEF; range: 1–30, higher scores are better), a PRO measure [13, 14]. Measures of effect included the response ratio, response difference, and odds ratio. For each measure of effect, the uncorrected (original) and corrected (adjusted) data were calculated using StatXact™ 11.1.0.

### Example 1: Erectile Function Domain Scores at Week 12

Based on a previous validation study, a cutoff score of 25 on the erectile function domain of the IIEF was determined to separate men classified as having normal erectile function (scores from 26 to 30 inclusive) versus men having less than normal erectile functioning (scores from 1 to 25 inclusive), which includes mild or more severe forms; the sensitivity and specificity of the IIEF erectile function domain were 0.97 and 0.88, respectively [15, 16]. Of interest here is the relationship between treatment groups (sildenafil vs. placebo) and whether or not normal erectile function was achieved at week 12 based on the erectile function domain of the IIEF (responder with a score from 26 to 30 versus non-responder otherwise).

It should be noted that successful treatment can be defined in different ways and can still be achieved without reaching as high a hurdle as normal erectile functioning at week 12. Other metrics for successful treatment, which do not require complete transition to normal erectile functioning, include to change favorably by at least a minimum amount, as illustrated next.

### Example 2: Change in Erectile Function Domain Scores at Week 12

Based on another validation study, at least a 4-point improvement was determined to be the “minimal clinically important difference” on the erectile function domain of the IIEF; the corresponding sensitivity and specificity values were 0.74 and 0.73, respectively [17]. In our example, an individual increase of at least four points on the erectile function domain from baseline to week 12 was considered as clinically meaningful within-patient improvement. Of interest here is the relationship between treatment groups (sildenafil vs. placebo) and whether or not an increase of at least four points was achieved on the change score of the erectile function domain (yes, for responder versus no, for non-responders).

## Results

### Example 1

Table 5 shows the unadjusted data of response by treatment where a responder is classified as having had a normal erectile function, defined as a score on the erectile function domain above 25 (26 to 30) at week 12. The unadjusted proportion who responded with sildenafil was 0.48 (= 54/112), while the unadjusted proportion who responded with placebo was 0.14 (= 16/115). Given sensitivity and specificity of response equal to 0.97 and 0.88, respectively, Table 5 also shows the corresponding corrected or adjusted data of response by treatment. The adjusted proportion who responded with sildenafil was 0.43 (= 47.7/112), while the adjusted proportion who responded with placebo was 0.02 (= 2.6/115).

**Table 5:** Example 1: Unadjusted data and adjusted data

Outcome status	Treatment		
	Sildenafil	Placebo	Total
Responder	54^a^	16	70
	47.7^b^	2.6	50.3
Non-responder	58	99	157
	64	112.4	176.7
Total	112	115	227
	112	115	227

*Note*: A responder had an erectile function domain score above 25 (26–30)

^a^The first entry in each cell is the observed (unadjusted) count of patients

^b^The second entry in each cell is the corrected (adjusted) count assuming sensitivity = 0.97 and specificity = 0.88

Based on Table 5, a comparison can be made between the unadjusted and adjusted results in terms of three metrics of treatment effects: response ratio, response difference, and odds ratio. The adjusted results indicated a much larger effect of active treatment for all three metrics; in this particular case, the unadjusted results underestimated the treatment effect. The estimated adjusted response ratio of 18.84 was much higher than the estimated unadjusted response ratio of 3.47 (95% confidence interval, CI: 2.15 to 5.71) and not even within the latter’s 95% CI. The estimated adjusted odds ratio of 32.07 was considerably much higher than the estimated odds ratio of 5.76 (95% CI: 3.02 to 10.98) and not even within 95% CI for the unadjusted odds ratio.

While the adjusted response difference of 0.41 is within the 95% confidence interval for the unadjusted response difference of 0.34 (95% CI 0.23 to 0.45), which does not necessarily imply lack of a statistical significance between the adjusted and unadjusted values, the difference between the two response differences of 0.07 (= 0.41–0.34) is noticeable (approximately one-fifth of the unadjusted response difference). In fact, *if* the adjusted proportion of 0.41 is taken as the fixed population proportion, a one-sample test for a proportion would give a statistically significant difference at the 0.05 level of significance between the adjusted and unadjusted response difference [*Z* = 0.07/square root of [(0.41)(0.59)/227] = 0.07/0.033 = 2.14 > 1.96].

### Example 2

Table 6 shows the unadjusted data of response by treatment where a responder is classified as having had at least a four-point increase or improvement in the erectile function domain from baseline to week 12. The unadjusted proportion who responded with sildenafil was 0.76 (= 85/112), while the unadjusted proportion who responded with placebo was 0.41 (= 47/115). Given sensitivity and specificity of response equal to 0.74 and 0.73, respectively, Table 6 (second cell entry) also shows the corresponding corrected (adjusted) data of response by treatment. Therefore, within-patient change is effectively determined by adjusting patient counts using the original responder threshold and diagnostic criteria (sensitivity and specificity), without adjusting the threshold itself**.**

**Table 6:** Example 2: Unadjusted and adjusted data

Outcome status	Treatment		
	Sildenafil	Placebo	Total
Responder	85^a^	47	
	116.5^b^	33.9	
	111.8^c^	32.6	
Non-responder	27	68	95
	− 4.5	81.1	76.6
	0.2	82.4	82.7
Total	112	115	227
	112	115	227
	112	115	27

*Note* A responder had an improvement in erectile function domain of at least four points

^a^The first entry in each cell is the observed (unadjusted) count of patients

^b^The second entry in each cell is the corrected (adjusted) count assuming sensitivity = 0.74 and specificity = 0.73

^c^The third entry in each cell is the corrected (adjusted) count assuming sensitivity = 0.76 and specificity = 0.73

But this table includes an expected corrected cell count that is negative, which is not permissible. What happened? When sensitivity < *a*/(*a* + *c*), as is the case here [0.74 < 85/(85 + 27) = 0.76], the corrected value for *C* becomes negative. An alternative approach is to choose the closest permissible value to the observed sensitivity so that this revised value of specificity equals [*a*/(*a* + *c*)] = 85/(85 + 27) = 0.76 instead of 0.74, with no change in specificity (0.73). Table 6 provides the revised corrected data of response by treatment. The revised adjusted proportion who responded with sildenafil became 0.998 (= 111.8/112), while the revised adjusted proportion who responded with placebo became 0.283 (= 32.6/115).

Based on Table 6, a comparison can be made between the unadjusted and (revised) adjusted results in terms of three metrics of treatment effects: response ratio, response difference, and odds ratio. The adjusted results indicated a much larger effect of treatment for all three metrics; in this particular case, the unadjusted results underestimated the treatment effect. The estimated adjusted response ratio of 3.53 was much higher than the estimated unadjusted response ratio of 1.86 (95% CI 1.47–2.39) and not within the 95% CI for the unadjusted response ratio. The estimated adjusted odds ratio of 1412.93 was vastly much higher than the estimated odds ratio of 4.56 (95% CI 2.57–8.06) and nowhere near the 95% CI for the unadjusted odds ratio. Moreover, the estimated adjusted response difference of 0.72 was twice as high as the estimated unadjusted response difference of 0.35 (95% CI 0.23–0.47) and not within the 95% CI for the unadjusted response ratio.

## Discussion

### Anchor-Based Methodology

Patient-reported measures, like all measurement instruments, are not perfectly reliable owing to measurement error [18, 19]. As subjective assessments, PRO measurement requires a series of judicious checks and balances.

Consider the context of change scores on a PRO measure to define a responder after treatment intervention. The concern here is that some individuals may be classified as responders (based on the cutoff or threshold score on the targeted PRO measure of interest) when in fact they have not changed according to an external indicator of true change taken as the “gold standard”; these individuals exhibit false-positive (observed) changes. Similarly, some individuals may be classified as non-responders (again based on the same cutoff or threshold score on the targeted PRO measure) when in fact they truly have changed according to the same external criterion; these individuals exhibit false-negative changes. The external criterion, which serves as the gold standard, is referred in the literature as an anchor measure, that is, a benchmark to define patients who have experienced a meaningful change in their condition [3, 9, 19–22].

Therefore, selecting the appropriate anchor measure(s) is of prime importance, as is the method for linking the anchor measure to the targeted PRO measure. The anchor indicator should be plainly understood in context, be easier to interpret than the PRO measure itself, and be sufficiently correlated to the targeted PRO measure. Anchor-based methods use the anchor criterion to determine what patients consider to be a presumably true meaningful change in their condition and relate changes on the PRO instrument to this criterion. Anchors that reflect degree of treatment benefit can be used to determine a threshold score on a PRO instrument for use as a responder definition [1]. Other considerations for selecting a suitable anchor indicator and a discussion of anchor-based methodology are given elsewhere [23, 24].

The topic of this article is simply a targeted extension and formalization of that found and accepted in conventional epidemiological settings for misclassification of outcome or disease—including what may be a patient-reported outcome—where adjustments in counts are needed to account for the measurement error in the subjective outcome. For instance, consider the binary outcome for self-reported peptic ulcer (yes, no) at the beginning and end of the study. Even though it is the subject who determines whether he or she has peptic ulcer (and therefore whether a change in peptic ulcer has occurred), each of the two assessments and therefore their change may be fraught with classification bias for multiple reasons and such measurement error would need to be addressed.

The same line of reasoning applies to a self-reported diagnostic test whose sum score is based on a series of questions and whose particular threshold score for disease vs. no disease (as the outcome) is based on a biopsy that serves as the gold standard. The sensitivity and specificity of the diagnostic test itself is expected, as an imperfect classification tool, to be fallible with measurement error (despite being based on patient response) and the resulting classification bias would need to be addressed.

As noted, the anchor method is used to determine the threshold that defines a responder on the targeted PRO measure of interest. While the subject’s self-report score relative to that threshold is central for defining response, the subject’s self-report score is not perfectly measured because the instrument upon which it is based is not a perfect indicator: The PRO instrument itself contains measurement error in the same way that diagnostic tests and self-report outcomes do in epidemiologic studies. In the two examples provided in the manuscript, only when the instrument’s sensitivity and specificity are each equal to 1 with respect to the external anchor criterion is there no classification or responder bias, with no calibration or adjustment needed.

Anchor-based methodology is used to determine sensitivity and specificity [1, 3, 9, 19–23], and the two IIEF examples featured in the manuscript use, in particular, a receiver operating characteristic (ROC) curve analysis to obtain sensitivity and specificity. For the example on erectile function (EF) domain scores (from the patient-reported IIEF) at week 12 (Example 1), sensitivity and specificity were obtained from previous validation research [15] where the anchor criterion was whether the patient had established clinical diagnosis of erectile dysfunction (ED). This dichotomy (yes, no) was regressed on the EF domain score, the targeted PRO measure of interest, in a logistic regression model and, using ROC curve analysis, the optimal cutoff score on the EF domain was selected essentially to maximize the average of sensitivity and specificity. Sensitivity and specificity were 0.97 and 0.88, respectively.

For the example given on change in EF domain scores at week 12 (Example 2), sensitivity and specificity were obtained from previous validation research [17] where the anchor was patient-reported level of satisfaction with sexual intercourse, also from the IIEF, selected because of its relevance to the US National Institutes of Health definition of ED and its grounding in prior psychometric research. Change scores, from baseline to week 12, on the satisfaction item were dichotomized into improvement or no improvement. This dichotomy was regressed on the change score of the EF domain, the targeted PRO measure of interest, in a logistic regression model and, using ROC curve analysis, the optimal cutoff on the change score of EF domain was selected based to maximize the sum of sensitivity and specificity (i.e., where most patients are correctly classified by the cutoff change score on the IIEF EF domain as having improved versus not having improved). Sensitivity and specificity were 0.74 and 0.73, respectively.

For subjective measures in general, the FDA recommends (also supported by the medical literature) three types of anchors, as external criteria approximating truth, to generate appropriate thresholds for meaningful within-patient change: (1) static, current-state global impression of severity scale (e.g., patient global impression of severity); (2) global impression of change scale (e.g., patient global impression of change); and (3) well-established clinical outcomes (if relevant) [24].

### Responder Analysis in Perspective

Responder analysis is of course not a new subject. Nor is adjustment for misclassification (information) bias or measurement error on exposure status or disease status or both. But the fusion of the two topics is novel in the context of clinical outcome assessments in general and PRO measures in particular. In this manuscript, correction formulas for misclassification bias on binary disease status (yes, no) are translated and used to correct for misclassification of binary responder status (yes, no) based on a PRO measure, with binary exposure status (experimental treatment, control treatment) is assumed to be measured perfectly. In a well-conducted randomized controlled trial the assumption of no misclassification in treatment status is quite reasonable.

The methodology in this paper centered on patient-reported measures as the focus point but is general enough to also apply to other types of clinical outcome assessments [25, 26] including clinician-reported outcome measures, observer-reported outcome measures, and performance outcome measures, as well as other outcomes beyond traditional disease status. Moreover, the methodology is applicable across therapeutic areas, extending beyond urology (featured in this article) and including (but not limited to) oncology where PROs have a major role in patient progression (where being a responder is an unfavorable rather than favorable outcome).

The value of enhancing interpretation of PRO measures using a responder definition based on meaningful within-person change, which naturally and inevitably leads to a comparison of responder rates (proportions) between treatment groups, has been discussed [1, 4]. Such a dichotomy serves as a practical, comprehensible way to distill complicated phenomena into simple categories [27]. Stakeholders of health can understand percent or proportion of success between treatment groups (such as a difference in proportions) as an intuitive, understandable metric of treatment benefit.

It should be emphasized, however, that the main analysis of patient-reported measures with quantitative (ordinal or continuous) data should be analyzed as such, rather than a dichotomized version of them, in order to preserve the full information and natural structure inherent in the original data [5, 24]. This article, therefore, does not advocate that the original metric of a quantitative PRO measure be replaced with a discretized version of it for the main analysis. On the contrary, for a PRO variable analyzed as continuous, the primary metric for treatment effect should be the difference in mean scores, or in mean change in baseline, emanating from a regression or longitudinal model for continuous data.

For instance, consider Example 2 on a responder analysis involving at least a four-point improvement from baseline on the erectile function domain scores at week 12. Its main analysis from an analysis of covariance model was prospectively based on the mean change from baseline between the sildenafil group [9.3 points; 95% confidence interval (CI), 7.9–10.7] and the placebo group (3.6 points; 95% CI 2.2–5.0) [12], which can be augmented by the results of the retrospective responder analysis presented and interpreted in this current paper. This difference of 5.7 points (95% CI 3.8–7.6; *p*-value < 0.001) is compatible or consonant with an adjusted response difference and ratio of 0.72 and 3.53, respectively (based on a response proportion of 0.998 from placebo minus response proportion of 0.283 from placebo). Thus, responder analysis is intended to supplement, not replace, such a main analysis for the purpose of advancing interpretation of a quantitative PRO measure above and beyond its primary analysis and interpretation from original data using a type of regression model [7, 8].

As long as the analytic plan for statistical inference for a quantitative PRO measure is pre-specified in the right order, with the analysis of means superseding the analysis of proportions, the interpretation of treatment effect using difference in mean scores and in responder proportions are not mutually exclusive. Both metrics of treatment effect can be complementary, coexisting synergistically, with the difference in responder proportion serving as an interpretive aid to augment the primary analysis based on difference in mean scores.

### Misclassification Issues

The same set of conclusions made about non-differential (and differential) disease misclassification in this article also apply to non-differential (and differential) responder misclassification, as the latter is a variant of the former. Non-differential responder misclassification occurs when the proportion of subjects misclassified on responder status does not depend on the status of the subject with respect to treatment status (or any other variables that might be in the analysis). Bias introduced by non-differential misclassification of a binary response, which like that of binary disease status of which it is variant, is predictable in being bias toward the null value of no treatment effect (provided that the misclassification is independent of other errors), as it is also for non-differential misclassification of a binary exposure status (like treatment) [28, 29]. Therefore, the clear underestimation of treatment effect (as measured by the response difference, response ratio, and odds ratio) in the two case studies is expected. By extension, as is the case for simultaneous (joint) misclassification of exposure and disease [29, 30], non-differential joint misclassification of both treatment and response will also generally result in bias toward from the null (provided no misclassification of covariates that might be presented in the analysis), if misclassification of dichotomous treatment is independent of dichotomous response status.

It should be noted, though, that non-differentially alone does not guarantee bias toward the null. While non-differential misclassification in most situations is expected or predicted to result in bias toward the null, non-differential response (or treatment) classification can at times produce bias away from the null if the response (or treatment) variable has more than two levels or if the classification errors depend on errors from other variables, as would also be the case more generally for exposure or disease misclassification [29, 30]. In contrast, differential misclassification of response status (like differential disease or exposure classification) causes unpredictable bias in the response difference, response ratio, or odd ratio that is either towards or away from the null, depending on the proportions of subjects misclassified [10, 11, 28, 29].

Misclassification of responders and non-responders stems from the imperfection of the PRO measure owing to multiple factors that may bias patients’ responses [31]. This article focuses on addressing bias for responder misclassification under the assumption of no treatment misclassification when there are two levels of responder status (yes, no) and of treatment status (experimental, control). In general, if errors in detecting the presence of a responder are equal between experimental treatment and control treatment (with sensitivity less than 100%), but no errors are made in the classification of non-responders (i.e., specificity is 100%), then the response ratio will not be biased but the odds ratio and risk difference will be biased towards the null value of no effect. If no errors are made in detecting the presence of a responder (i.e., 100% sensitivity), but equal errors are made among treatment and comparison groups in the classification of non-responders (with specificity less than 100%), then response ratio, odds ratio and response difference will be biased toward the null.

As stated and illustrated in this article, when there is no misclassification of treatment, a limitation of the formulas intended to correct for non-differential misclassification of binary responder status may yield negative and hence inappropriate results for the corrected cell frequencies. Following from the same set of circumstances and limitations as correcting for non-differential misclassification of binary disease status [10], adjusting for non-differential misclassification of binary responder status gives a negative corrected count in any one of five situations [where sensitivity (SE) and specificity (SP) are for misclassification of responder status]: (1) SE + SP = 1; (2) SP < *c*/(*a* + *c*), giving a negative corrected value for *A*; 3) SP < *d*/(*b* + *d*), giving a negative corrected value for *B*; (4) SE < *a*/(*a* + *c*), giving a negative corrected value for *C* (which was found in the second example); and (5) SE < *b*/(*b* + *d*), giving a negative corrected value for *D*. Although not a perfect solution, one viable way to address this problem is to select the closest alternative value to SE or SP that changes a cell count from negative to positive, as was performed in the second example. Further research is encouraged to investigate negative cell counts and how to best address them.

The simple bias-correction analysis introduced here for responder analysis of PRO measures is an improvement over its conventional counterpart, which implicitly assumes no misclassification error at all on responder status (100% sensitivity and 100% specificity). But this simple bias-correction implies that the diagnostic parameters (i.e., sensitivity and specificity) are fixed and known without error, a situation that is rarely realized. For example, there is no bona fide gold standard of measurement for responder status; the chosen anchor measure may be suitable approximation for true responder status but not a perfect, error-free indicator of it. Thus there is expected uncertainty in the sensitivity and specificity rates themselves. This limitation is not restricted to PRO measures but applies generally to many exposure and outcome variables in epidemiology [11].

During the validation stage of quantifying misclassification rates for responder status, the use and concordance of multiple anchor measures is one way to mitigate the lack of an undisputed gold standard of responder status. As with the case of epidemiologic outcomes, responder status for PRO measures can extend beyond the simple bias-correction analysis to, for instance, multidimensional bias analysis (where the methods for simple bias-correction analysis are repeated with a plausible range of values for sensitivity and specificity) and probabilistic bias analysis (where probability distributions are assigned to sensitivity and specificity that, after Monte Carlo sampling techniques, generate a frequency distribution of correct estimates of effect) [11], which can also allow for differential misclassification of responder status between treatments. While these more advanced methods are beyond the scope of this article, they deserve attention for addressing responder analysis of PRO measures. In addition, cumulative distribution functions, one for each treatment group, can be used to evaluate a range of responder cutoffs on a PRO measure and thereby assess the robustness of the chosen cutoff [1, 3, 4].

One area of research on misclassification issues involves expanding classification from a dichotomy of response (responder, no responder) to a trichotomy (improvement, stable, deterioration) and correcting for misclassification bias. These more discriminating responder categorizations in a longitudinal study are expected to be more sensitive and discerning than a simple responder dichotomy in reflecting the main analysis on the original continuous scale of an instrument. In oncology, for instance, such a candidate instrument for three-level responder adjustment includes the European Organization for Research and Treatment of Cancer Quality of Life Questionnaire that has been analyzed longitudinally based on its original metric in the primary analysis and also to classify subjects with at least a 10-point improvement, at least 10-point deterioration, and otherwise no real change (stable) in secondary analyses [32].

## Conclusion

In summary, quantitative PRO measures ideally are analyzed on their original metric in the primary analysis and, as a way to convey meaning and understanding of PRO scores, responder analyses are often used in a secondary analysis to complement the interpretation of those primary analyses. Thus, a useful way to lend meaning and interpretation to a quantitative PRO measure is to dichotomize between values where within-patient changes are considered clinically important and those that are not. Nonetheless, a PRO scale with a cutoff score that discriminates well between responders and non-responders is still typically fraught with misclassification of responder status, at least to a certain extent, while there is usually no treatment misclassification in a well-conducted study.

The methodology in this article can be applied to different therapeutic areas and different types of clinical outcome assessments. In the context of PRO measures, this article provides formulas that correct for responder misclassification under the assumption of no treatment misclassification and illustrates the methodology with two case studies from sexual medicine. As such, treatment effect bias from misclassification of responder status on PRO measures is addressed and corrected, leading to more trustworthy interpretation and effective decision-making.

## Data Availability

Upon request, and subject to certain criteria, conditions, and exceptions (see https://www.pfizer.com/science/clinical-trials/trial-data-and-results for more information), Pfizer will provide access to individual de-identified participant data from Pfizer-sponsored global interventional clinical studies conducted for medicines, vaccines, and medical devices (1) for indications that have been approved in the US and/or EU or (2) in programs that have been terminated (i.e., development for all indications has been discontinued). Pfizer will also consider requests for the protocol, data dictionary, and statistical analysis plan. Data may be requested from Pfizer trials 24 months after study completion. The de-identified participant data will be made available to researchers whose proposals meet the research criteria and other conditions, and for which an exception does not apply, via a secure portal. To gain access, data requestors must enter into a data access agreement with Pfizer.
